# Impact of concomitant left-sided valve disease on outcomes following tricuspid valve transcatheter edge-to-edge repair: insights from EuroTR

**DOI:** 10.1093/eschf/xvag103

**Published:** 2026-05-20

**Authors:** Jonas Gmeiner, Lukas Stolz, Karl-Patrik Kresoja, Jennifer von Stein, Vera Fortmeier, Christoph Pauschinger, Wolfgang Rottbauer, Mohammad Kassar, Bjoern Goebel, Paolo Denti, Paul Achouh, Tienush Rassaf, Manuel Barreiro-Perez, Peter Boekstegers, Andreas Rück, Monika Zdanyte, Marianna Adamo, Flavien Vincent, Philipp Schlegel, Sebastian Rosch, Mirjam G Wild, Christian Besler, Stefan Toggweiler, Stephanie Brunner, Julia Grapsa, Tiffany Patterson, Holger Thiele, Tobias Kister, Giuseppe Tarantini, Giulia Masiero, Marco De Carlo, Alessandro Sticchi, Fabian Voss, Amin Polzin, Antonio Popolo Rubbio, Francesco Bedogni, Thorald Stolte, Thomas Nestelberger, Thomas Benito-Gonzales, Enrique Sánchez-Muñóz, Mathias H Konstandin, Eric Van Belle, Marco Metra, Tobias Geisler, Rodrigo Estévez-Loureiro, Amir Abbas Mahabadi, Nicole Karam, Francesco Maisano, Philipp Lauten, Fabien Praz, Mirjam Kessler, Daniel Kalbacher, Volker Rudolph, Christos Iliadis, Philipp Lurz, Jörg Hausleiter, the EuroTR Investigators, Julia Novotny, Ludwig T Weckbach, Thomas J Stocker, Kaspar Volz, Katalin Berschiminski, Philipp Doldi, Hannah Kempton, Dario Grassini, Clemens Scherer, Karl-Philip Rommel, Ralph Stephan von Bardeleben, Roman Pfister, Stephan Baldus, Philipp von Stein, Muhammed Gerçek, Felix Rudolph, Sebastian Ludwig, Benedikt Koell, Leonhard-Moritz Schneider, Dominik Felbel, Carsten Salomon, Harald Lapp, Quentin de Baynast, Alain Berrebi, Florian Schindhelm, Berenice Caneiro-Queija, Julio Echarte-Morales, Andreas Goldschmied, Edoardo Pancaldi, Elisa Pezzola, Mauro Massussi, Laura Lupi, Natacha Rousse, Samy Aghezzaf, Norbert Frey, Martin Kraus, Dirk Westermann, Federico Arturi, Anke von Peter

**Affiliations:** Medizinische Klinik und Poliklinik I, LMU Klinikum, LMU München, Munich, Germany; Medizinische Klinik und Poliklinik I, LMU Klinikum, LMU München, Munich, Germany; German Center for Cardiovascular Research (DZHK), Partner Site Munich Heart Alliance, Munich, Germany; Department of Cardiology, Cardiology I, University Medical Center of the Johannes Gutenberg-University Mainz, Mainz, Germany; Department of Cardiology, Heart Center, University of Cologne, Cologne, Germany; Clinic for General and Interventional Cardiology/Angiology, Herz- und Diabeteszentrum NRW, Universitätsklinik der Ruhr-Universität Bochum, Med. Fakultät OWL (Universität Bielefeld), Bad Oeynhausen, Germany; Department of Cardiology, University Heart and Vascular Centre Hamburg, Hamburg, Germany; German Center of Cardiovascular Research (DZHK), Partner Site Hamburg/Kiel/Lübeck, Germany; Ulm University Heart Center, Ulm, Germany; Clinic for General and Interventional Cardiology/Angiology, Herz- und Diabeteszentrum NRW, Universitätsklinik der Ruhr-Universität Bochum, Med. Fakultät OWL (Universität Bielefeld), Bad Oeynhausen, Germany; Department of Cardiology, Inselspital, Bern University Hospital, University of Bern, Bern, Switzerland; Graduate School for Health Sciences, University of Bern, Switzerland; Department of Cardiology, Heart Center, Zentralklinik Bad Berka, Robert-Koch-Allee 9, Bad Berka 99437, Germany; Heart Valve Center, Cardio-Thoracic-Vascular Department, IRCCS San Raffaele Scientific Institute, Milan, Italy; Cardiology Department, European Hospital Georges Pompidou, Université Paris Cité, Paris, France; Department of Cardiology and Vascular Medicine, West German Heart and Vascular Center, University Hospital Essen, University of Duisburg-Essen, Essen, Germany; Hospital Álvaro Cunqueiro, Vigo, Spain; Department of Cardiology, Helios Klinikum Siegburg, Siegburg, Germany; Department of Cardiology, Karolinska University Hospital, Stockholm, Sweden; Medical Clinic III, University Hospital Tübingen, Tübingen, Germany; ASST Spedali Civili di Brescia and Department of Medical and Surgical Specialties, Radiological Sciences, and Public Health, University of Brescia, Brescia, Italy; Cardiology Department, Centre Hospitalier Universitaire De Lille, Lille, France; Department of Internal Medicine III, Division of Cardiology, University Hospital Heidelberg, Ruprecht-Karl University Heidelberg, Heidelberg 69120, Germany; Department of Cardiology, Cardiology I, University Medical Center of the Johannes Gutenberg-University Mainz, Mainz, Germany; Department of Cardiology and Angiology, Medical Center, Faculty of Medicine, University Heart Center, University of Freiburg, Hugstetter Str. 55, 79106, Freiburg, Germany; Department of Cardiology and Angiology, Medical Center, Faculty of Medicine, University Heart Center, University of Freiburg, Hugstetter Str. 55, 79106, Freiburg, Germany; Heart Center Lucerne, Luzerner Kantonsspital, Lucerne, Switzerland; Heart Center Lucerne, Luzerner Kantonsspital, Lucerne, Switzerland; Department of Cardiology, Guys and St Thomas NHS Trust, London, UK; Department of Cardiology, Guys and St Thomas NHS Trust, London, UK; Department of Cardiology, Heart Center Leipzig at Leipzig University, Leipzig, Germany; Department of Cardiology, Heart Center Leipzig at Leipzig University, Leipzig, Germany; Department of Cardiac, Thoracic Vascular Sciences and Public Health, University of Padua, Padua, Italy; Department of Cardiac, Thoracic Vascular Sciences and Public Health, University of Padua, Padua, Italy; University of Pisa, Pisa, Italy; University of Pisa, Pisa, Italy; Department of Cardiology, Pulmonology and Angiology, Medical Faculty, Heinrich Heine University of Düsseldorf, Düsseldorf 40225, Germany; Department of Cardiology, Pulmonology and Angiology, Medical Faculty, Heinrich Heine University of Düsseldorf, Düsseldorf 40225, Germany; Department of Cardiology, IRCCS Policlinico San Donato, San Donato Milanese, Milan, Italy; Department of Cardiology, IRCCS Policlinico San Donato, San Donato Milanese, Milan, Italy; Cardiovascular Research Institute Basel (CRIB) and Department of Cardiology, University Hospital Basel, University of Basel, Basel, Switzerland; Cardiovascular Research Institute Basel (CRIB) and Department of Cardiology, University Hospital Basel, University of Basel, Basel, Switzerland; Departamento de Cardiología, Complejo Asistencial Universitario de León, León, Spain; Departamento de Cardiología, Complejo Asistencial Universitario de León, León, Spain; Department of Internal Medicine III, Division of Cardiology, University Hospital Heidelberg, Ruprecht-Karl University Heidelberg, Heidelberg 69120, Germany; Cardiology Department, Centre Hospitalier Universitaire De Lille, Lille, France; ASST Spedali Civili di Brescia and Department of Medical and Surgical Specialties, Radiological Sciences, and Public Health, University of Brescia, Brescia, Italy; Medical Clinic III, University Hospital Tübingen, Tübingen, Germany; Hospital Álvaro Cunqueiro, Vigo, Spain; Department of Cardiology and Vascular Medicine, West German Heart and Vascular Center, University Hospital Essen, University of Duisburg-Essen, Essen, Germany; Cardiology Department, European Hospital Georges Pompidou, Université Paris Cité, Paris, France; Heart Valve Center, Cardio-Thoracic-Vascular Department, IRCCS San Raffaele Scientific Institute, Milan, Italy; Department of Cardiology, Heart Center, Zentralklinik Bad Berka, Robert-Koch-Allee 9, Bad Berka 99437, Germany; Department of Cardiology, Inselspital, Bern University Hospital, University of Bern, Bern, Switzerland; Ulm University Heart Center, Ulm, Germany; Department of Cardiology, University Heart and Vascular Centre Hamburg, Hamburg, Germany; German Center of Cardiovascular Research (DZHK), Partner Site Hamburg/Kiel/Lübeck, Germany; Clinic for General and Interventional Cardiology/Angiology, Herz- und Diabeteszentrum NRW, Universitätsklinik der Ruhr-Universität Bochum, Med. Fakultät OWL (Universität Bielefeld), Bad Oeynhausen, Germany; Department of Cardiology, Heart Center, University of Cologne, Cologne, Germany; Department of Cardiology, Cardiology I, University Medical Center of the Johannes Gutenberg-University Mainz, Mainz, Germany; Medizinische Klinik und Poliklinik I, LMU Klinikum, LMU München, Munich, Germany; German Center for Cardiovascular Research (DZHK), Partner Site Munich Heart Alliance, Munich, Germany

**Keywords:** Multivalvular disease, Aortic regurgitation, Aortic stenosis, Mitral regurgitation, Mitral stenosis, Tricuspid regurgitation

## Abstract

**Introduction:**

The impact of coexisting left-sided valvular heart disease (VHD) on clinical outcomes following tricuspid valve edge-to-edge repair (T-TEER) for tricuspid regurgitation (TR) remains unclear, particularly under real-world conditions. To evaluate the prevalence and prognostic impact of concomitant left-sided VHD in patients undergoing T-TEER.

**Methods:**

This study included all patients undergoing T-TEER from the European Registry of Transcatheter Repair for Tricuspid Regurgitation (EuroTR; NCT06307262) with complete echocardiographic data on left-sided valve disease. Study endpoints included survival and heart failure hospitalizations (HFH) at 2 years, NYHA functional class, and TR reduction.

**Results:**

Among a total of 1647 eligible patients, 95.8%, 35.6%, and 3.8% had ≥mild, moderate, and severe concomitant VHD, respectively. Moderate or higher VHD was associated with a significantly reduced 2-year survival (*P* < .001) and reduced 2-year HFH-free survival (*P* = .005). Multivariate regression analysis confirmed ≥ moderate VHD to be an independent predictor of mortality (hazard ratio 1.54, 95% CI 1.21–1.96, *P* < .001). Despite worse TR and NYHA functional class at baseline in patients with ≥moderate VHD, T-TEER was associated with a significant TR reduction (*P* < .001) and symptomatic improvement (*P* < .001).

**Conclusion:**

Concomitant left-sided VHD is common among patients undergoing T-TEER and is independently associated with worse survival and higher rates of HFH. Nevertheless, T-TEER provides meaningful symptomatic benefit and durable TR reduction in patients with and without VHD burden.

## Graphical Abstract

**Graphical Abstract:**
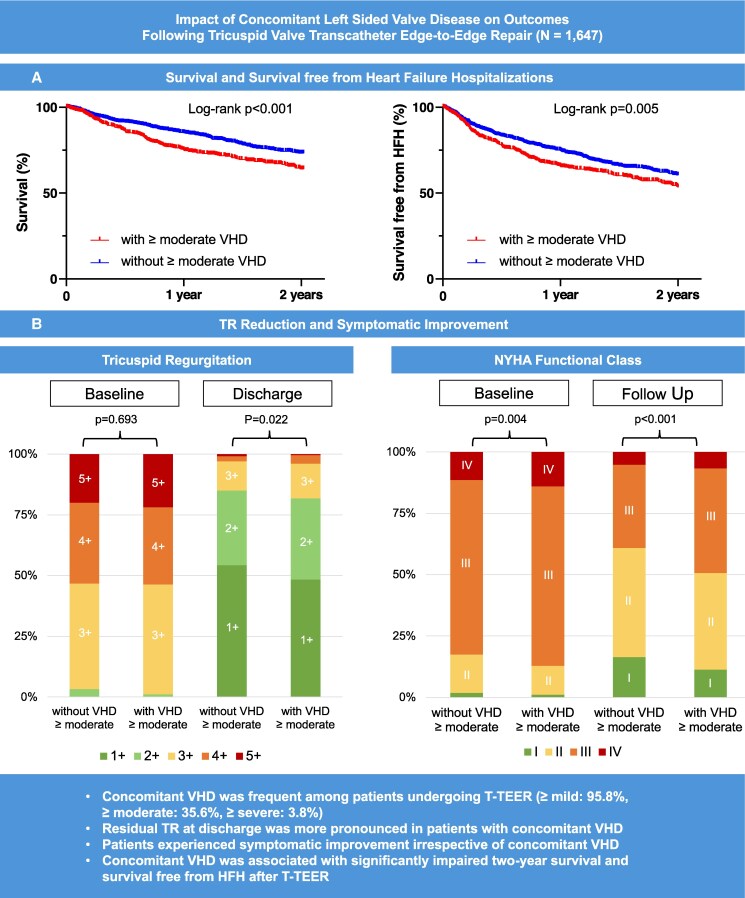

## Introduction

Tricuspid regurgitation (TR) is associated with considerable morbidity and mortality, as well as a marked impairment in quality of life.^1^ Historically, the limited availability of low-risk treatment options contributed to the underappreciation of TR in clinical practice. In recent years, tricuspid valve transcatheter edge-to-edge repair (T-TEER) has gained recognition as a safe, less-invasive, and efficacious therapeutic option for patients with clinically relevant TR.^2,3^ In the randomized controlled TRILUMINATE and Tri.FR trials, T-TEER led to a significant improvement in quality of life compared to optimal medical therapy alone.^4,5^ Beyond that, recently published 2-year follow-up data reported a reduction in the cumulative incidence of heart failure hospitalization (HFH) for patients undergoing T-TEER.^6^ However, the TRILUMINATE and Tri.Fr trials imposed strict inclusion criteria, among other comorbidities, leading to the exclusion of patients with relevant concomitant left-sided valve disease.^7^ Under real-world conditions, many patients present with concomitant disease of the mitral or aortic valve, but data on the prognostic impact of multivalvular heart disease in the setting of T-TEER are scarce and mainly limited to patients with severe mitral regurgitation or aortic stenosis presenting with concomitant TR.^8,9^ To our knowledge, no large multicentre registry has comprehensively assessed the independent prognostic impact of thoroughly graded left-sided valvular disease across the full severity spectrum in T-TEER patients. Therefore, this study aimed at assessing the prevalence as well as the clinical impact of coexisting left-sided valve disorders in patients undergoing T-TEER, utilizing data from the large international European Registry of Transcatheter Repair for Tricuspid Regurgitation (EuroTR).

## Methods

### Study cohort and procedural technique

The ongoing EuroTR comprises patients who underwent T-TEER for symptomatic TR between 2016 and 2024. Details regarding the registry have previously been published.^10–12^ For the current analysis, individuals were excluded if they had undergone concomitant mitral TEER (M-TEER) or if echocardiographic data on concomitant left-sided valve disease severity were missing (Supplementary Table S1).

At the time of evaluation, all patients continued to experience symptoms despite receiving the maximally tolerated doses of diuretic therapy. Treatment decisions were made following assessment by a multidisciplinary heart team, typically composed of specialists in heart failure, cardiac surgery, and interventional cardiology.

T-TEER procedures were performed using either the PASCAL system (Edwards Lifesciences, Irvine, CA, USA) or the MitraClip/TriClip system (Abbott, Santa Clara, CA, USA). The study was conducted in compliance with the Declaration of Helsinki and received appropriate ethical approval (NCT06307262).

### Study variables and endpoints

Baseline clinical variables encompassed demographic data (age and sex), prevalent comorbidities such as systemic arterial hypertension, prior myocardial infarction, coronary artery disease, history of stroke, and diabetes mellitus. Symptoms related to heart failure were systematically recorded, including dyspnoea as classified by the New York Heart Association (NYHA) functional scale, peripheral oedema, ascites, jugular venous distension, and the presence of pleural effusion.

Echocardiographic assessments were conducted in accordance with established guideline recommendations using the effective regurgitation orifice area, and biplane vena contracta measurement was used whenever possible.^13,14^ TR severity was assessed using a five-grade scale^15^: mild (1+), moderate (2+), severe (3+), massive (4+), and torrential (5+). Mitral valve regurgitation was assessed using a four-grade scale, while all other valve disease was assessed using a three-grade scale.

The cohort was divided into patients with or without concomitant ≥ moderate valvular heart disease. The primary endpoint of the study was all-cause mortality at 2 years. Secondary endpoints included survival free from heart failure-related hospitalizations and changes in NYHA functional class at the most recent clinical follow-up. Subgroup analysis assessed patients with concomitant VHD of mild, moderate, and severe degrees, as well as individual left-sided valve disease. Systematic echocardiographic follow-up of left-sided valve lesions (MR, AS, AR, and MS) was not available across the registry and could therefore not be incorporated into the present analysis.

### Statistical analysis

Statistical analysis was performed using IBM SPSS (version 28.0.0.0). Normally distributed continuous variables were reported as mean ± standard deviation, non-normally distributed continuous variables as median with interquartile ranges, and categorical variables were reported as percentages. Students' *t*-test, Mann-Whitney U test, and Wilcoxon test were used to compare groups as appropriate. All tests were two-tailed, and *P*-values <.05 were considered significant. Mortality rates were calculated using the Kaplan–Meier method, comparisons were made by log-rank tests, and Kaplan–Meier curves were plotted using GraphPad Prism version 8.

## Results

### Baseline characteristics and overall outcomes

**Figure 1:**
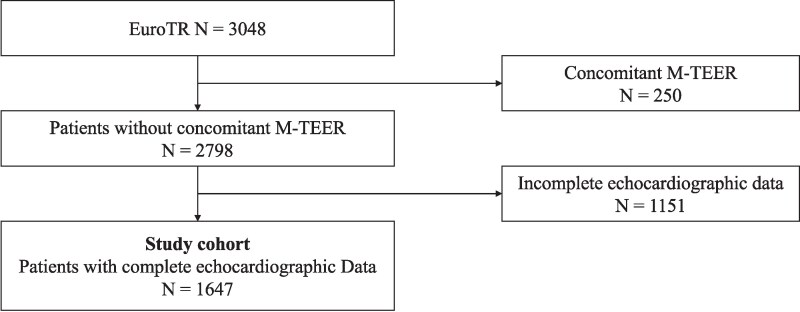
Study flowchart. The figure shows the study flowchart. Patients undergoing M-TEER and patients with incomplete echocardiographic data were excluded from this analysis. M-TEER = mitral transcatheter-edge-to-edge-repair

**Figure 2:**
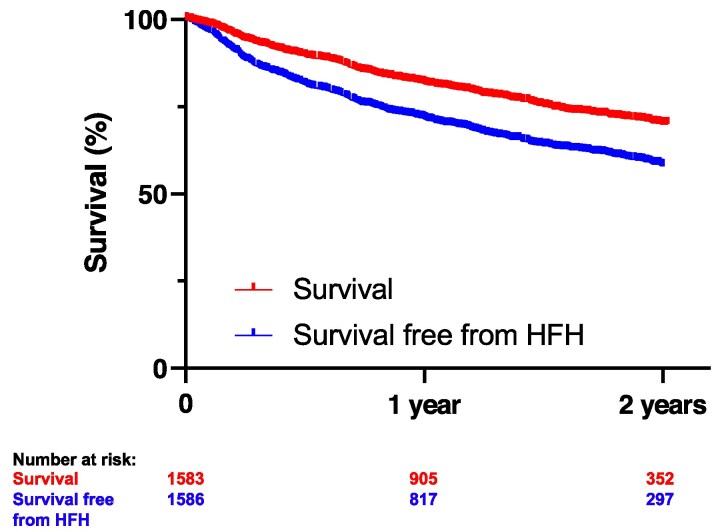
Kaplan–Meier curves for survival and survival free from heart failure hospitalizations of the total study cohort. Kaplan–Meier curves show the 2-year survival and survival free from heart failure hospitalizations() of the total study cohort. HFH = heart failure hospitalizations

Among a total of 3048 patients in the EuroTR, 1647 patients did not undergo concomitant M-TEER and had complete echocardiographic data on concomitant left-sided valve disease and were therefore included in the present analysis (*Figure 1*). Baseline characteristics of the study cohort are presented in *Table 1*. Patients exhibited advanced surgical risk as indicated by the EuroScore II (6.5 ± 7.2%) and TRI-Score (5.7 ± 2.0) and were predominantly female (54.1%), with a preserved left ventricular ejection fraction (53.0 ± 10.9%). TR was successfully reduced to ≤ moderate and ≤mild in 74.8% and 31.9% of patients, respectively. At the 2-year follow-up, all-cause survival and survival free from HFH were 70.4% and 58.7% (*Figure 2*). T-TEER was associated with a significant improvement in the NYHA functional class and durable reduction of TR at the latest available follow-up (Supplementary Figure S1). Left ventricular stroke volume increased significantly between baseline and the latest available follow-up (47.5 (37.0–58.1) ml vs. 49.0 (38.0–60.7) ml, *P* = .006).

**Table 1:** Baseline characteristics of patients with and without concomitant ≥ moderate VHD

	Total cohort *N* = 1647	Without concomitant ≥ moderate VHD *n* = 1061	With concomitant ≥ moderate VHD *n* = 586	*P*-value*
Age (years)	78.8 ± 7.8	78.6 ± 8.3	79.4 ± 6.8	**.026**
BMI (kg/m^2^)	25.0 (22.4–28.3)	25.2 (22.7–28.5)	24.6 (22.0–27.7)	**<**.**001**
EuroScore II	6.5 ± 7.2	6.4 ± 7.0	6.7 ± 7.4	.408
TRI-Score	5.7 ± 2.0	5.5 ± 2.0	5.9 ± 1.9	.**006**
Male sex	756 (45.9)	506 (47.4)	250 (42.7)	.056
H/o myocardial infarction	141 (10.1)	96 (10.5)	45 (9.4)	.487
COPD	236 (15.2)	151 (15.0)	85 (15.7)	.692
PAD	180 (13.0)	120 (13.2)	60 (12.5)	.727
Diabetes mellitus	179 (21.7)	228 (21.8)	122 (21.6)	.903
H/o stroke	179 (12.7)	115 (12.5)	64 (13.1)	.759
H/o cardiac surgery	395 (25.4)	252 (24.9)	143 (26.4)	.529
H/o tricuspid valve surgery	18 (1.3)	15 (1.6)	3 (0.6)	.100
Right ventricular lead	470 (28.6)	295 (27.8)	175 (29.9)	.364
Atrial fibrillation	1469 (90.8)	941 (90.0)	528 (92.3)	.132
CAD	695 (43.1)	441 (42.3)	254 (44.5)	.393
NYHA functional class				.**004**
I	25 (1.5)	19 (1.8)	6 (1.0)	
II	234 (14.3)	167 (15.9)	67 (11.5)	
III	1181 (72.2)	750 (71.3)	431 (73.9)	
IV	195 (11.9)	116 (11.0)	79 (13.6)	
**Echocardiographic data**				
TR severity				.693
Moderate	38 (2.3)	32 (3.0)	6 (1.0)	
Severe	721 (43.9)	456 (43.1)	265 (45.3)	
Massive	538 (32.7)	350 (33.1)	188 (32.1)	
Torrential	347 (21.1)	221 (20.9)	126 (21.5)	
LVEF (%)	53 ± 11	53 ± 11	52 ± 11	.113
LVEDD (mm)	48.3 ± 8.6	47.9 ± 8.5	48.9 ± 8.7	.**029**
LA diameter	105.3 ± 56.3	103.3 ± 53.4	108.9 ± 61.1	.083
TR EROA (cm^2^)	0.58 (0.41–0.79)	0.59 (0.42–0.80)	0.56 (0.40–0.79)	.143
TR Vena contracta (mm)	11.2 ± 4.5	11.2 ± 4.8	11.3 ± 4.4	.491
RV EDA (cm^2^)	26.5 ± 9.6	26.9 ± 8.9	25.7 ± 10.8	.**039**
RV ESA (cm^2^)	16.4 ± 6.8	16.7 ± 6.7	15.9 ± 7.1	.073
RV mid (mm)	39.1 ± 8.9	39.8 ± 8.7	37.9 ± 9.2	**<**.**001**
RV basal (mm)	49.0 ± 12.7	49.5 ± 9.1	48.3 ± 8.8	.**011**
TV annular diameter (mm)	43.3 ± 8.1	43.2 ± 8.2	43.3 ± 8.1	.617
RAA (cm^2^)	34 (28–42)	34 (27–43)	34 (28–43)	.213
TAPSE (mm)	17.4 ± 4.6	17.5 ± 4.5	17.2 ± 4.6	.311
RAP (mmHg)	11.7 ± 6.7	11.6 ± 6.9	11.7 ± 6.1	.945
sPAP_echo_ (mmHg)	43.4 ± 14.1	42.1 ± 13.4	45.9 ± 15.0	**<**.**001**
Coaptation gap (mm)	6.2 ± 3.1	6.1 ± 2.9	6.4 ± 3.5	.190
Tenting height (mm)	7.6 ± 3.3	7.6 ± 3.4	7.7 ± 3.3	.579
Tenting area (cm^2^)	1.8 ± 1.1	1.8 ± 1.1	1.8 ± 1.1	.722
TR etiology				.750
Primary	198 (13.4)	130 (13.7)	68 (12.9)	
Secondary	1141 (77.3)	729 (76.7)	412 (78.3)	
Mixed	138 (9.3)	92 (9.7)	46 (8.7)	
**Medication**				
MRA	669 (44.3)	419 (42.3)	250 (48.4)	.**025**
Loop diuretics	1398 (92.7)	906 (91.6)	492 (94.8)	.**028**
Thiazide diuretics	338 (22.6)	223 (22.8)	115 (22.3)	.897
Betablocker	1259 (83.4)	817 (82.4)	442 (85.5)	.245
RAAS-inhibitor	835 (55.6)	560 (56.7)	275 (53.5)	.251
SGLT2-inhibitor	389 (26.7)	265 (27.7)	124 (24.8)	.262

Values are depicted as mean ± standard deviation, median (interquartile range) or number (percentage) as appropriate. Bold printed values indicated statisitcal significance (p<.005).

CAD, coronary artery disease; COPD, chronic obstructive pulmonary disease; EDA, end-diastolic area; EROA, effective regurgitant orifice area; ESA, end-systolic area; EuroSCORE, European System for Cardiac Operative Risk Evaluation; H/o , history of; LVEDD, left ventricular end-diastolic diameter; LVEF, left ventricular ejection fraction; MRA, mineralocorticoid rezeptor antagonist; PAD, peripheral arterial disease; RAA, right atrial area; RAAS, Renin–angiotensin–aldosterone system; RegVol, regurgitant volume; RV , right ventricular; RVbase, right ventricular basal diameter; RVmid, right ventricular midventricular diameter; sPAPecho, echocardiographically estimated systolic pulmonary artery pressure; TAPSE, tricuspid annular plane systolic excursion; TR, tricuspid regurgitation; TV, tricuspid valve; VC, vena contracta; VHD, valvular heart disease.

^*^ *P*-value for comparison of patients with and without concomitant VHD ≥ II°.

### Concomitant left-sided valve disease

Concomitant VHD of any degree was frequent among patients in the EuroTR (*Table 2*) but predominantly due to mild mitral (60.2%) and aortic regurgitation (38.5%). Additionally, 24.8% of patients had moderate mitral regurgitation, whereas other moderate VHD and severe valvular pathologies were infrequent among patients undergoing T-TEER after exclusion of patients undergoing concomitant M-TEER. In total, 1061 patients (64.4%) presented with concomitant ≥ moderate VHD. Only 3.8% of patients had severe left-sided VHD, precluding separate fully adjusted survival analyses for this subgroup. Regarding baseline characteristics, patients with concomitant ≥ moderate VHD exhibited an older age (78.6 ± 8.3 years vs. 79.4 ± 6.8 years, *P* = .026), lower body mass index (BMI: 25.2 [22.7–28.5] kg/m2 vs. 24.6 [22.0–27.7] kg/m2, *P* < .001), and higher TRI score (5.5 ± 2.0 vs. 5.9 ± 1.9, *P* = .006) than patients without concomitant ≥ moderate VHD (*Table 1*). Patients with concomitant ≥ moderate VHD had more dilated right ventricles and an increased estimated systolic artery pressure (*Table 1*).

**Table 2:** Prevalence of valvular heart disease

Severity	TR	MR	MS	AR	AS	Any concomitant VHD*
none	—	142 (8.6)	1525 (92.6)	922 (56.0)	1440 (87.4)	69 (4.2)
1°	—	1052 (63.9)	102 (6.2)	634 (38.5)	116 (7.0)	1578 (95.8)
2°	38 (2.3)	409 (24.8)	20 (1.2)	87 (5.3)	76 (4.6)	586 (35.6)
3°	721 (43.9)	43 (2.6)	—	4 (0.2)	15 (0.9)	62 (3.8)
4°	538 (32.7)	1 (0.1)	—	—	—	1 (0.1)
5°	347 (21.1)	—	—	—	—	

AR, aortic regurgitation; AS, aortic stenosis; MR, mitral regurgitation; MS, mitral stenosis; TR, tricuspid regurgitation; VHD, valvular heart disease.

^*^Equal or greater than.

### Impact of concomitant left-sided valve disease on survival, heart failure hospitalizations, and symptomatic outcomes

**Figure 3:**
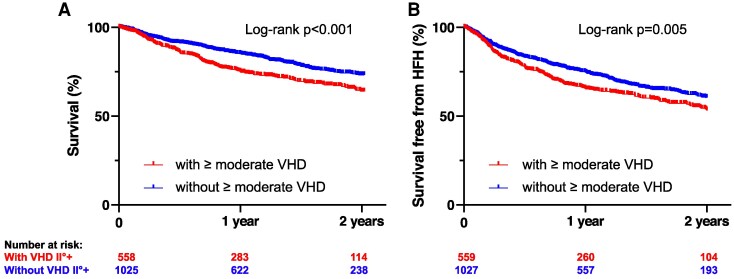
Kaplan–Meier curves of survival (*A*) and survival free from heart failure hospitalizations (*B*) of patients with and without concomitant ≥ moderate VHD. Kaplan–Meier curves show significantly reduced 2-year survival (*A*) and survival free from heart failure hospitalizations (*B*) of patients with ≥moderate VHD. HFH = heart failure hospitalizations; VHD = valvular heart disease

Concomitant ≥ moderate VHD was associated with a significantly reduced 2-year survival rate (*P* < .001, *Figure 3A*) and reduced HFH-free survival (*P* = .005, *Figure 3B*). Supplementary Figure S2 illustrates the Kaplan–Meier curves for survival and survival free from heart failure hospitalizations for patients with concomitant mild, moderate, and severe left-sided VHD. Supplementary Figure S3 presents lesion-specific Kaplan–Meier curves for the main left-sided valve entities (MR, AS, AR, and MS), indicating that the composite ≥ moderate VHD signal is predominantly driven by MR, whereas interpretation for the less frequent lesions is limited by small numbers. Multivariate Cox regression analysis identified concomitant ≥ moderate VHD, age, emergency intervention, male sex, LVEF, and TAPSE as independent predictors of survival (*Table 3*).

**Table 3:** Cox regression analysis

	Univariate			Multivariate		
	Hazard ratio	CI	*P*-value	Hazard ratio	CI	*P*-value
Concomitant VHD ≥ 2	1,51	1.22–1.84	**<.001**	1.51	1.18–1.92	**<**.**001**
Age (years)	1.00	0.99–1.02	.720			
Male sex	1.64	1.32–2.04	**<**.**001**	1.54	1.20–1.98	**<**.**001**
COPD	1.27	0.96–1.67	.097			
Diabetes mellitus	1.18	0.92–1.52	.183			
H/o cardiac surgery	1.42	1.13–1.80	.**003**	1.14	0.87–1.48	.33
H/o stroke	1.14	0.82–1.58	.434			
Emergency intervention	3.38	1.60–7.15	.**001**	2.88	1.35–6.15	.**006**
Atrial fibrillation	0.78	0.56–1.10	.155			
Coronary artery disease	1.45	1.17–1.80	**<**.**001**	1.11	0.87–1.43	.390
LVEF	0.98	0.97–0.98	**<**.**001**	0.99	0.98–1.00	.**026**
TAPSE	0.92	0.90–0.95	**<**.**001**	0.94	0.91–0.97	**<**.**001**
sPAP_echo_	1.01	1.00–1.02	.065			
RV FAC	1.00	0.99–1.01	.380			
Postprocedural TR	1.37	1.22–1.53	**<**.**001**	1.34	1.18–1.52	**<**.**001**

Bold printed values indicated statisitcal significance (p<.005).

H/o, history of; LVEF, left ventricular ejection fraction; TAPSE, tricuspid annular plane systolic excursion; RV FAC, right ventricular fractional area change; sPAP_echo_, echocardiographically estimated systolic pulmonary artery pressure; VHD, valvular heart disease.

**Figure 4:**
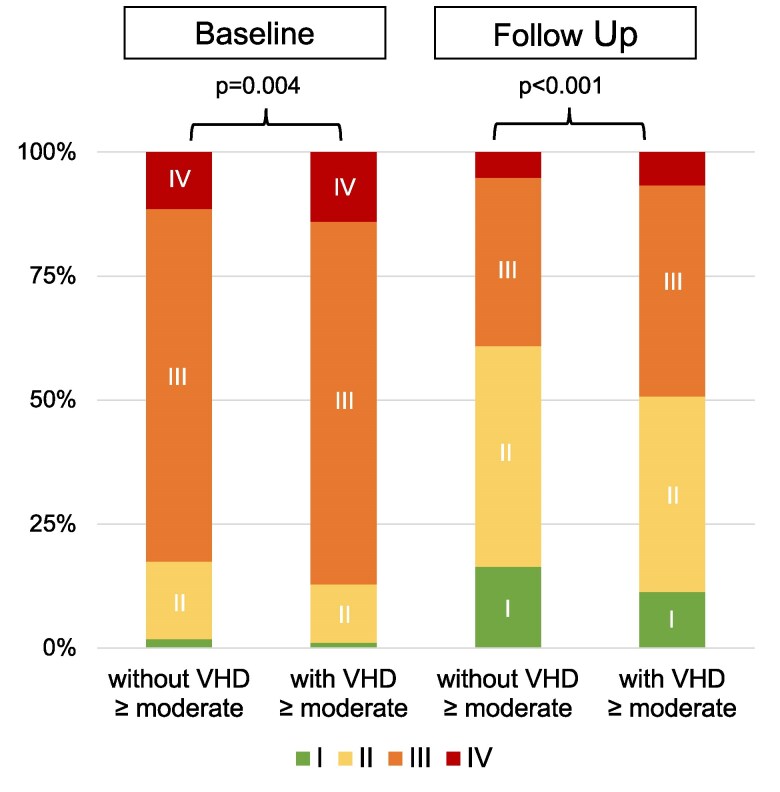
Pre- and post-procedural NYHA functional class of patients with and without concomitant VHD ≥ 2. The figure shows significant differences of NYHA functional class between patients with and without concomitant moderate valvular heart disease (VHD) before and after T-TEER

*Figure 4* illustrates the NYHA functional class at baseline and the latest available follow-up. Patients with concomitant ≥ moderate VHD had significantly worse NYHA functional class compared to patients without concomitant ≥ moderate VHD before and after T-TEER (*P* = .004 and *P* < .001, respectively). However, both groups showed significant improvement in NYHA functional class after T-TEER (*P* < .001).

**Figure 5:**
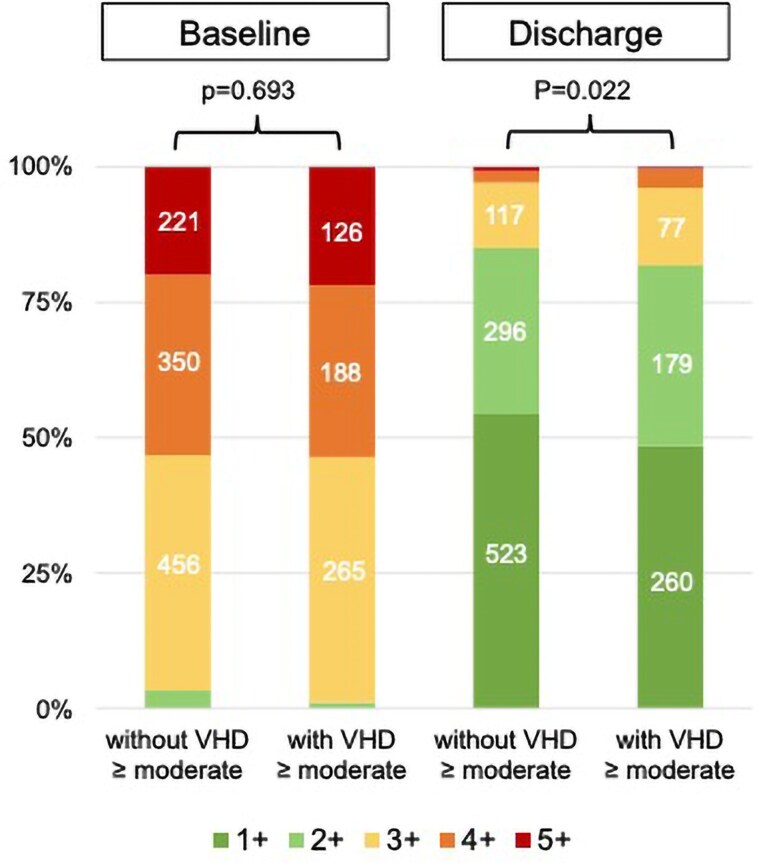
Pre- and post-procedural TR severity of patients with and without ≥ moderate VHD. The figure shows significant differences of TR severity between patients with and without concomitant moderate valvular heart disease (VHD) after T-TEER

Pre- and post-procedural severities of TR of both groups are outlined in *Figure 5*. Baseline TR did not differ significantly between groups (*P* = .693). Although both groups showed significant improvement in TR (*P* < .001), patients with concomitant ≥ moderate VHD had a higher degree of residual TR at discharge (*P* = .022).

## Discussion

The EuroTR is the largest cohort of patients treated with T-TEER for TR to date, comprising more than 3000 patients. Using this comprehensive database, the current analysis reports for the first time the prevalence and impact of concomitant left-sided valvular heart disease in patients undergoing T-TEER. The main findings of the present analysis can be summarized as follows:

1. Concomitant VHD was frequent among patients undergoing T-TEER (≥ mild: 95.8%, ≥ moderate: 35.6%, ≥ severe: 3.8%).
2. Residual TR at discharge was more pronounced in patients with concomitant VHD.
3. Irrespective of concomitant VHD, patients experienced symptomatic improvement.
4. Concomitant VHD was associated with significantly impaired 2-year survival and survival free from HFH after T-TEER.

Importantly, our analysis uniquely demonstrates that even moderate left-sided VHD independently predicts worse survival. This finding has direct clinical implications for patient selection and risk stratification, although concomitant VHD in this setting may act not only as a pathophysiologic contributor but also as a marker of an overall higher-risk phenotype.

Recently, the first randomized controlled TRILUMINATE trial showed that T-TEER improves quality of life and reduces heart failure hospitalizations.^5,6^ However, despite encouraging results from multiple registries, no mortality benefit could be demonstrated, which might be attributable to differences in patient populations.^7^ Real-world cohorts, including the present analysis, report that patients treated with T-TEER are of higher surgical risk, that is, present with a greater burden of comorbidities, such as atrial fibrillation, pulmonary hypertension, and more severe right ventricular dysfunction.^7^ Accordingly, the mortality observed in our cohort was higher compared to the TRILUMINATE trial, reflecting a more comorbid patient population. Importantly, our analysis highlights that this difference in baseline risk also applies to concomitant valvular heart disease. While the TRILUMINATE and Tri-Fr trials excluded patients with significant left-sided valve disease and did not report on mild concomitant valvular heart disease, we could demonstrate that more than one-third of ‘real-world’ T-TEER patients exhibit concomitant moderate VHD.

As expected, the most frequently observed valvular disease was mitral regurgitation, likely related to atrial dilation contributing to both atrial functional MR and TR. Additionally, a subset of patients presented with moderate aortic stenosis and regurgitation or mitral stenosis, as expected in an elderly patient population with the associated risk factors. Untreated severe left-sided valve disease was uncommon, reflecting contemporary clinical practice, in which these conditions are typically addressed before treatment of TR is considered, including concomitant M-TEER, which was excluded from this analysis.^16,17^

Treatment of patients with multivalvular disease is challenging. A complex interplay of cardiac haemodynamics complicates severity grading, and most trials have focused on single valvular diseases. Furthermore, despite the high prevalence of multivalvular disease among patients with severe valvular disease, guideline recommendations on multivalvular disease are scarce.^18^ These patients more often undergo additional diagnostic testing and are less frequently referred for intervention.^18^

To date, data on TR in the setting of multivalvular disease have largely been restricted to populations with severe left-sided valve disease and concomitant TR. In patients undergoing mitral TEER or transcatheter aortic valve implantation (TAVI), moderate to severe TR was common and consistently associated with worse long-term outcomes, including survival.^19–22^ Conversely, treatment of left-sided valve disease was frequently associated with a reduction in TR severity.^20,21^ Hence, TR was historically regarded mainly as a consequence of left-sided valve disease rather than a distinct clinical entity. Supporting this view, Genereux et al. proposed a new classification system for aortic stenosis grading beyond the echocardiographically obtained gradients.^23^ This system is based on the theory that even earlier stages of left-sided valve disease can cause upstream damage due to increased filling pressures and pulmonary hypertension, ultimately resulting in TR and right ventricular failure at advanced stages.

Our findings partially endorse this concept. Patients with ≥moderate left-sided valve disease exhibited higher systolic pulmonary artery pressure. Furthermore, we demonstrated that even moderate concomitant left-sided valve disease was associated with increased mortality, HFH, more severe symptoms, and lower procedural success. Nevertheless, T-TEER resulted in a significant and sustained reduction of TR and meaningful symptomatic improvement at 2 years, even in patients with relevant concomitant VHD. This challenges the notion that TR is merely a bystander phenomenon secondary to left-sided valve disease. Instead, TR itself may contribute to systemic congestion, end-organ dysfunction due to hypoperfusion, and right ventricular failure with reduced cardiac output—all of which can, in turn, exacerbate left-sided valve dysfunction. Recent studies have shown that T-TEER alters left-sided haemodynamics and improves MR in a significant percentage of patients.^24,25^ In our patient cohort, left ventricular stroke volume increased significantly after T-TEER. These findings suggest a bidirectional interplay between right- and left-sided valve disease, rather than a unidirectional upstream effect. Of note, echocardiographic follow-up data of concomitant valve disease were not available at the time of manuscript preparation. Therefore, we cannot disentangle the relative contributions of residual TR, progression, or improvement in left-sided VHD and other comorbidities to the observed outcomes, and the proposed interplay between right- and left-sided valve disease should be considered hypothesis-generating.

The higher residual TR in patients with concomitant VHD may be explained by different mechanistic concepts as follows: chronic left-sided valve disease maintains elevated left atrial and pulmonary pressures, which we demonstrated in our cohort. This may perpetuate right ventricular afterload elevation, impair RV–PA coupling, and consequently limit favourable geometric remodelling despite successful T-TEER. Greater left ventricular dimensions we found in patients with concomitant left-sided VHD might reflect left-sided disease unaffected by tricuspid repair. Conversely, larger RV dimensions in patients without left-sided VHD may suggest a TR phenotype favourable for procedural success.

The results of this study once again emphasize the importance of a comprehensive assessment of TR patients in a multidisciplinary heart team consisting of interventional cardiologists, surgeons, heart failure specialists, and electrophysiologists. Concomitant cardiac and extracardiac pathologies must be addressed as completely as possible to maximize the benefit of tricuspid valve interventions. In patients undergoing cardiac surgery, valve repair or replacement should be as complete as reasonably achievable to minimize the risk of repeat surgery. For transcatheter therapies, retrospective data have shown that after M-TEER, the rate of concomitant TR ≥2 + reduces around 10% (56% to 45%).^26^ However, predicting a respective improvement remains challenging. In a propensity-matched analysis from the TRAMI and TRIVALVE registry, concomitant M- and T-TEER was associated with better survival compared with isolated M-TEER in patients with both MR and TR.^8^ Nevertheless, randomized controlled studies are required in order to be able to provide a clear recommendation regarding simultaneous treatment of MR and TR.

Further studies are needed to not only report the impact of concomitant baseline VHD on outcomes after T-TEER but also investigate the potential changes in concomitant valvular pathologies after TR treatment.

### Study limitations

Despite using data from the largest multicentre registry of patients undergoing T-TEER, this study has inherent limitations. Data collection was based on site-reported information without confirmation by an independent echocardiographic core laboratory, which represents a critical limitation. VHD grading—particularly moderate lesions—is operator-dependent and prone to interobserver variability, with no systematic variability data available in EuroTR. Misclassification may have biased estimates in either direction; hence, the results require cautious interpretation and validation in core lab-adjudicated cohorts. A subset of patients lacked complete echocardiographic data and were therefore excluded. At the time of manuscript preparation, follow-up echocardiographic data on concomitant valve disease were not available, representing a major limitation of the study, as it prevented assessment of temporal changes of concomitant VHD and therefore restricts mechanistic interpretation. Patients with concomitant moderate VHD showed slightly higher utilization of diuretics at baseline, but these differences likely reflect disease severity rather than differential therapeutic strategies confounding the prognostic signal. Furthermore, patients with ≥moderate left-sided VHD had higher TRI-score, more advanced RV remodelling, and higher pulmonary pressures at baseline, indicating more severe global cardiopulmonary disease. Although we adjusted our multivariable models for several clinical and echocardiographic covariates, residual confounding cannot be excluded, and concomitant left-sided VHD in this cohort may represent a marker of advanced cardiopulmonary disease rather than an isolated causal determinant of outcome. While lesion-specific Kaplan–Meier curves are shown in Supplementary Figure S3, the low prevalence of non-MR lesions precluded fully adjusted models for each valve entity, limiting generalizability beyond MR.

## Conclusion

In the largest T-TEER registry to date, patients presented with a high prevalence of multivalvular disease. Concomitant left-sided valve disease was associated with increased mortality and heart failure hospitalizations. T-TEER was associated with significant TR reduction and symptomatic improvement regardless of concomitant valve disease, but given the observational design and absence of follow-up imaging, these findings must be interpreted cautiously. Prospective studies are needed to validate these observations and to clarify the mechanistic interplay between right- and left-sided valve disease.

## Supplementary Material

xvag103_Supplementary_Data

## Declarations

### Data Availability

No data were generated or analysed for this manuscript.

### Pre-registered Clinical Trial Number

None supplied.

### Appendix

**‡PubMed indexed investigator list:** Julia Novotny (Medizinische Klinik und Poliklinik I, LMU Klinikum, LMU München, Munich, Germany), Ludwig T. Weckbach (Medizinische Klinik und Poliklinik I, LMU Klinikum, LMU München, Munich, Germany; Cardiovascular Research Institute Basel (CRIB) and Department of Cardiology, University Hospital Basel, University of Basel, Switzerland), Thomas J. Stocker (Medizinische Klinik und Poliklinik I, LMU Klinikum, LMU München, Munich; Cardiovascular Research Institute Basel (CRIB) and Department of Cardiology, University Hospital Basel, University of Basel, Switzerland), Kaspar Volz (Medizinische Klinik und Poliklinik I, LMU Klinikum, LMU München, Munich), Katalin Berschiminski (Medizinische Klinik und Poliklinik I, LMU Klinikum, LMU München, Munich), Philipp Doldi (Medizinische Klinik und Poliklinik I, LMU Klinikum, LMU München, Munich), Hannah Kempton (Medizinische Klinik und Poliklinik I, LMU Klinikum, LMU München, Munich; Clinic for General and Interventional Cardiology/Angiology, Herz- und Diabeteszentrum NRW, Universitätsklinik der Ruhr-Universität Bochum, Med. Fakultät OWL (Universität Bielefeld), Bad Oeynhausen, Germany), Dario Grassini (Medizinische Klinik und Poliklinik I, LMU Klinikum, LMU München, Munich; German Center for Cardiovascular Research (DZHK), Partner Site Munich Heart Alliance, Munich, Germany), Clemens Scherer (Medizinische Klinik und Poliklinik I, LMU Klinikum, LMU München, Munich), Karl-Philip Rommel (Department of Cardiology, Cardiology I, University Medical Center of the Johannes Gutenberg-University Mainz, Mainz, Germany), Ralph Stephan von Bardeleben (Department of Cardiology, Cardiology I, University Medical Center of the Johannes Gutenberg-University Mainz, Mainz, Germany), Roman Pfister (Department of Cardiology, Heart Center, University of Cologne, Cologne, Germany), Stephan Baldus (Department of Cardiology, Heart Center, University of Cologne, Cologne, Germany), Philipp von Stein (Department of Cardiology, Heart Center, University of Cologne, Cologne, Germany), Muhammed Gerçek (Clinic for General and Interventional Cardiology/Angiology, Herz- und Diabeteszentrum NRW, Universitätsklinik der Ruhr-Universität Bochum, Med. Fakultät OWL (Universität Bielefeld), Bad Oeynhausen, Germany), Felix Rudolph (Department of Cardiology, Heart Center, University of Cologne, Cologne, Germany), Sebastian Ludwig (Department of Cardiology, University Heart and Vascular Centre Hamburg, Hamburg, Germany; German Center of Cardiovascular Research (DZHK), Partner Site Hamburg/Kiel/Lübeck, Germany), Benedikt Koell (Department of Cardiology, University Heart and Vascular Centre Hamburg, Hamburg, Germany; German Center of Cardiovascular Research (DZHK), Partner Site Hamburg/Kiel/Lübeck, Germany), Leonhard-Moritz Schneider (Ulm University Heart Center, Ulm, Germany), Dominik Felbel (Ulm University Heart Center, Ulm, Germany), Carsten Salomon (Department of Cardiology, Heart Center, Zentralklinik Bad Berka, Robert-Koch-Allee 9, Bad Berka 99437, Germany), Harald Lapp (Department of Cardiology, Heart Center, Zentralklinik Bad Berka, Robert-Koch-Allee 9, Bad Berka 99437, Germany), Quentin de Baynast (Cardiology Department, European Hospital Georges Pompidou, Université Paris Cité, Paris, France), Alain Berrebi (Cardiology Department, European Hospital Georges Pompidou, Université Paris Cité, Paris, France), Florian Schindhelm (Department of Cardiology and Vascular Medicine, West German Heart and Vascular Center, University Hospital Essen, University of Duisburg-Essen, Essen, Germany), Berenice Caneiro-Queija (Hospital Álvaro Cunqueiro, Vigo, Spain), Julio Echarte-Morales (Hospital Álvaro Cunqueiro, Vigo, Spain), Andreas Goldschmied (Medical Clinic III, University Hospital Tübingen, Tübingen, Germany), Edoardo Pancaldi (ASST Spedali Civili di Brescia and Department of Medical and Surgical Specialties, Radiological Sciences, and Public Health, University of Brescia, Brescia, Italy), Elisa Pezzola (ASST Spedali Civili di Brescia and Department of Medical and Surgical Specialties, Radiological Sciences, and Public Health, University of Brescia, Brescia, Italy), Mauro Massussi (ASST Spedali Civili di Brescia and Department of Medical and Surgical Specialties, Radiological Sciences, and Public Health, University of Brescia, Brescia, Italy), Laura Lupi (ASST Spedali Civili di Brescia and Department of Medical and Surgical Specialties, Radiological Sciences, and Public Health, University of Brescia, Brescia, Italy), Natacha Rousse (Cardiology Department, Centre Hospitalier Universitaire De Lille, Lille, France), Samy Aghezzaf (Cardiology Department, Centre Hospitalier Universitaire De Lille, Lille, France), Norbert Frey (Department of Internal Medicine III, Division of Cardiology, University Hospital Heidelberg, Ruprecht-Karl University Heidelberg, Heidelberg 69120, Germany), Martin Kraus (Department of Internal Medicine III, Division of Cardiology, University Hospital Heidelberg, Ruprecht-Karl University Heidelberg, Heidelberg 69120, Germany), Dirk Westermann (University Heart Center Freiburg/Bad Krozingen, Bad Krozingen, Germany), Federico Arturi (Department of Cardiac, Thoracic Vascular Sciences and Public Health, University of Padua, Padua, Italy), Anke von Peter (Cardiovascular Research Institute Basel (CRIB) and Department of Cardiology, University Hospital Basel, University of Basel, Switzerland).
