# Supplementary material for: Impact of concomitant left-sided valve disease on outcomes following tricuspid valve transcatheter edge-to-edge repair: insights from EuroTR
Source: ESC Heart Fail. 2026 May 20;13(3):xvag103. doi: 10.1093/eschf/xvag103 (PMC13202459; doi:10.1093/eschf/xvag103)
Supplement: xvag103_Supplementary_Data [file xvag103_supplementary_data.docx]

Supplemental Table 1: Baseline characteristics of excluded patients

| n = 1647 | Included patients (n=1647) | Excluded patients (n=1401) | p-value |
| --- | --- | --- | --- |
| Age (years) | 78.8±7.8 | 78.5±7.5 | 0.121 |
| BMI (kg/m2) | 25.0 (22.4 – 28.3) | 25.2 (22.7 – 28.8) | 0.137 |
| LVEF (%) | 53.0±10.9 | 52.7 ±11.3 | 0.546 |
| EuroScore II | 6.5±7.2 | 6.2±5.7 | 0.187 |
| TRI-Score | 5.7±2.0 | 6.0±1.9 | **0.004** |
| Male sex | 45.9% | 45.3% | 0.734 |
| H/o myocardial infarction | 10.1% | 11.5% | 0.237 |
| COPD | 15.2% | 16.0% | 0.565 |
| PAD | 13.0% | 10.1% | 0.096 |
| Diabetes mellitus | 21.7% | 25.6% | **0.017** |
| H/o stroke | 12.7% | 10.2% | 0.071 |
| H/o cardiac surgery | 25.4% | 29.3% | **0.019** |
| H/o tricuspid valve surgery | 1.3% | 1.8% | 0.289 |
| Right ventricular lead | 28.6% | 26.8% | 0.291 |
| Atrial fibrillation | 90.8% | 89.4% | 0.197 |
| Coronary artery disease | 43.1% | 39.3% | **0.036** |

Supplemental Figure 1: Improvement of NYHA functional class and TR reduction of the total study cohort


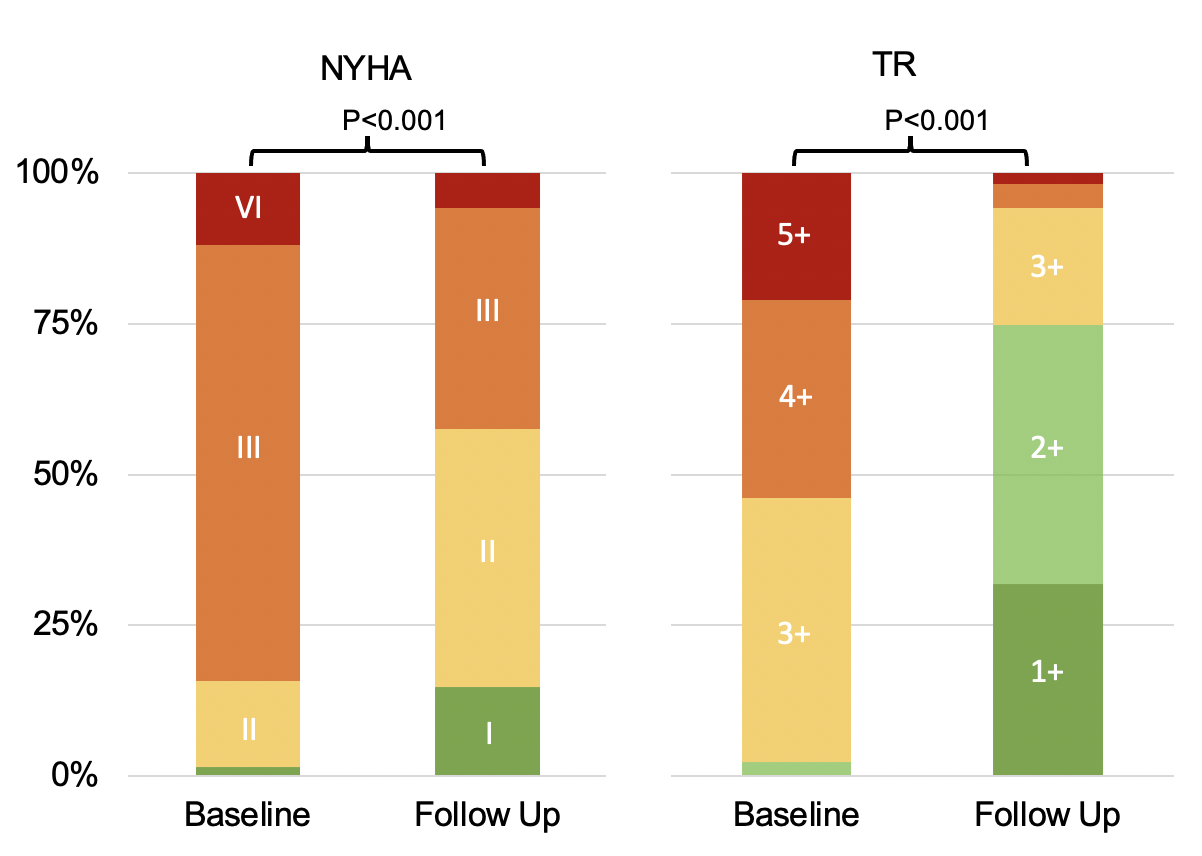


TR = tricuspid regurgitation

Supplemental Figure 2: Kaplan Meier curves for survival and survival free from heart failure hospitalizations for patients with concomitant left sided valvular disease 1, 2, and 3.

HFH = heart failure hospitalizations

VHD = valvular heart disease

Supplemental Figure 3: Kaplan Meier curves of distinct left sided valve disease

AR = aortic regurgitation

AS = aortic stenosis

MR = mitral regurgitation

MS = mitral stenosis
